# Comparison of Arterial Spin Labeling and Dynamic Susceptibility Contrast Perfusion MRI in Patients with Acute Stroke

**DOI:** 10.1371/journal.pone.0069085

**Published:** 2013-07-16

**Authors:** Yen-Chu Huang, Ho-Ling Liu, Jiann-Der Lee, Jen-Tsung Yang, Hsu-Huei Weng, Meng Lee, Mei-Yu Yeh, Yuan-Hsiung Tsai

**Affiliations:** 1 Department of Neurology, Chang Gung Memorial Hospital at Chiayi, Chang-Gung University College of Medicine, Tao-Yuan, Taiwan; 2 Department of Diagnostic Radiology, Chang Gung Memorial Hospital at Chiayi, Chang-Gung University College of Medicine, Tao-Yuan, Taiwan; 3 Department of Neurosurgery, Chang Gung Memorial Hospital at Chiayi, Chang-Gung University College of Medicine, Tao-Yuan, Taiwan; 4 Department of Biomedical Imaging and Radiological Sciences, National Yang Ming University, Taipei, Taiwan; 5 Department of Medical Imaging and Radiological Sciences, Chang Gung University, Tao-Yuan, Taiwan; Institution of Automation, CAS, China

## Abstract

**Background:**

The aim of this study was to evaluate whether arterial spin labeling (ASL) perfusion magnetic resonance imaging (MRI) can reliably quantify perfusion deficit as compared to dynamic susceptibility contrast (DSC) perfusion MRI.

**Methods:**

Thirty-nine patients with acute ischemic stroke in the anterior circulation territory were recruited. All underwent ASL and DSC MRI perfusion scans within 30 hours after stroke onset and 31 patients underwent follow-up MRI scans. ASL cerebral blood flow (CBF) and DSC time to maximum (T_max_) maps were used to calculate the perfusion defects. The ASL CBF lesion volume was compared to the DSC T_max_ lesion volume by Pearson's correlation coefficient and likewise the ASL CBF and DSC T_max_ lesion volumes were compared to the final infarct sizes respectively. A repeated measures analysis of variance and least significant difference post hoc test was used to compare the mean lesion volumes among ASL CBF, DSC T_max_ >4–6 s and final infarct.

**Results:**

Mean patient age was 72.6 years. The average time from stroke onset to MRI was 13.9 hours. The ASL lesion volume showed significant correlation with the DSC lesion volume for T_max_ >4, 5 and 6 s (*r* = 0.81, 0.82 and 0.80; *p*<0.001). However, the mean lesion volume of ASL (50.1 ml) was significantly larger than those for T_max_ >5 s (29.2 ml, *p*<0.01) and T_max_ >6 s (21.8 ml, *p*<0.001), while the mean lesion volumes for T_max_ >5 or 6 s were close to mean final infarct size.

**Conclusion:**

Quantitative measurement of ASL perfusion is well correlated with DSC perfusion. However, ASL perfusion may overestimate the perfusion defects and therefore further refinement of the true penumbra threshold and improved ASL technique are necessary before applying ASL in therapeutic trials.

## Introduction

In patients with acute ischemic stroke, magnetic resonance imaging (MRI) is a sensitive tool used to detect the perfusion abnormalities. The mismatch between the infarct core on diffusion-weighted imaging (DWI) and the hypoperfused region on perfusion-weighted imaging (PWI) may indicate potentially salvageable cerebral ischemic tissue [1]. This concept has been applied to select patients for thrombolytic therapy in acute stroke beyond 3 hours [2], [3], [4]. Until now, all clinical trials have relied on dynamic susceptibility contrast (DSC) to evaluate hypoperfused areas in acute ischemic stroke. However, DSC perfusion requires the administration of intravenous gadolinium contrast, which is contraindicated in patients with renal insufficiency or allergy to gadolinium excluding patients who may benefit from thrombolytic therapy.

Arterial spin-labeling (ASL) is an alternative MRI technique that can be used to identify and quantify hypoperfused areas. Perfusion imaging with ASL uses magnetically labeled arterial blood water protons as endogenous tracer particles. The major advantage of this method is that it provides noninvasive, quantitative measurements of cerebral blood flow (CBF) with relative insensitivity to permeability. In addition, it does not require administration of contrast agents, and it allows repeated measurements that may be used to evaluate multiple interventions [5]. In earlier studies, ASL, like DSC perfusion, was found to assess perfusion abnormalities in acute ischemic stroke [6], [7], [8], and it was sensitive enough to detect perfusion defects in small infarcts and transient ischemic attacks as well [9], [10]. ASL could enable quantitative measurement of relative CBF in the core and mismatch regions [11], and it was used to depict large perfusion defects in agreement with DSC MRI [12], [13]. However, it is uncertain whether ASL accurately depicts the area of perfusion abnormality and presumably identifies the penumbra in acute stroke as compared to DSC.

The aim of the present study is to evaluate whether as compared to DSC perfusion, ASL perfusion can quantify perfusion deficit consistently.

## Materials and Methods

### Patients

This prospective study was conducted from December 2010 to July 2012. Patient inclusion criteria were: (1) acute ischemic stroke without thrombolytic therapy, (2) the complete MRI protocol (described later) performed within 30 hours after onset of symptoms, and (3) presence of a visible lesion on DWI within the anterior or middle cerebral artery territories. The final infarct size was estimated by follow-up MRI for 31 patients. Patients were excluded if their glomerular filtration rate (GFR) was less than 30 ml/minute per 1.73 m2. The study was performed under a protocol approved by the Institutional Review Board of Chang Gung Memorial Hospital, and all examinations were performed after obtaining written informed consent.

### Imaging

#### MRI instrumentation and procedures

All data were collected using a 3 Tesla Siemens Verio MRI system (Siemens Medical System, Erlangen, Germany) using a 16 channel head coil. The first MRI protocol included axial DWI, MR angiography, ASL, and DSC perfusion imaging; the follow-up protocol included DWI, MR angiography and fluid-attenuated inversion recovery (FLAIR) imaging.

The ASL scans were performed using a pulsed ASL pulse sequence, Q2TIPS (TI_1_  = 600 m s, TI_2_  = 1600 ms, field of view (FOV)  = 220 mm, matrix  = 64×64, 15×6-mm slices, GRAPPA acceleration with iPAT  = 2, repetition time (TR)  = 3 seconds, echo time (TE)  = 11 ms, NEX  = 61 with 30 pairs of tags and controls acquired in 3 min 14 s). The DSC perfusion weighted imaging scans were acquired using a gradient echo EPI sequence (TR  = 1500 ms, TE  = 36 ms, FOV  = 220 mm, matrix  = 92×92, 17×6-mm slices, and scan time  = 1 min 36 s) with an intravenous bolus injection of gadolinium contrast agent (0.2 mmol/kg) at the fifth dynamic. DW MR imaging was performed using a EPI sequence with b = 0 and b = 1000 s/mm^2^, in three dimensions in space to result in four images per section. The imaging parameters used for this were TR/TE  = 5600/93 ms, acquisition matrix of 130×130, 4.0 mm slice thickness, and a 230-mm FOV. http://asist.umin.jp/data-e.shtmlDW images were processed to generate pixel-by pixel trace apparent diffusion coefficient (ADC) imaging. The MR angiography used three-dimensional (3D) time of flight (TR/TE  = 21/3.6 ms and 0.6 mm thickness) covering the extracranial carotid artery and the circle of Willis. The FLAIR sequence included 28 contiguous axial oblique slices (TR/TE  = 9000/85 ms, TI  = 2500 ms, 165×256 acquisition matrix, 4.0 mm slice thickness, and 230 mm FOV).

#### Postprocessing and Image Analysis

Perfusion Mismatch Analyzer (PMA, Ver.3.4.0.6, ASIST, Japan) [14] was used to calculate the DSC perfusion imaging for each patient. PMA software is developed for analyzing computed tomography and MR perfusion images [15], [16], [17]. The quantitative time to maximum (T_max_) maps were generated using standard singular value decomposition. The PMA software selected 5 to 10 pixel for arterial input function (AIF) automatically, based on histograms of peak concentration, time-to-peak and mean transit time. If the selected the pixels were not in adjacent to the middle cerebral artery (MCA), or the concentration time curves exhibited apparent saturation effect, the pixels were deleted manually. The T_max_ perfusion maps for 4, 5, and 6 s delay thresholds were used to estimate the perfusion deficit volumes in each patient using semi-automated local software programs. The infarct core volume was measured within ADC imaging [18]. The final infarct size was measured within FLAIR imaging [19].

The ASL CBF maps were generated on the scanner console, which were calculated using the following equation [20]:
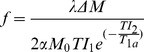
Where Δ*M* is the difference signal between tag and control acquisitions, *M*_0_ is the equilibrium magnetization, λis the blood/tissue water partition coefficient (0.9), *T*_1a_ is the longitudinal relaxation time of blood (1496 ms), αis the inversion efficiency (0.95). The CBF maps were evaluated by an experienced stroke neurologist (Y.C.H) and a neuroradiologist (Y.H.T.), both blinded to the DSC perfusion data. Both readers were allowed to see the DWI and ADC images for reference and reached a consensus on the ASL perfusion defect criteria before reading. The ASL CBF perfusion defects, which were defined by comparing the lesion site to the corresponding contralateral healthy brain, were selected visually for each patient by the 2 readers independently.

### Statistical analysis

All statistical analyses were performed using the Statistical Program for Social Sciences (SPSS) statistical software (version 18, Chicago, IL, USA). Data were expressed as mean±standard deviation. An intraclass correlation coefficient (ICC) in single measure, two-way mixed model, was used to examine the reliability between the 2 image readers (Y.C.H and Y.H.T.) in evaluating the ASL CBF maps. The average ASL CBF volume was compared to each DSC perfusion volume in T_max_ maps for 4, 5, and 6 s delay by Pearson's correlation coefficient, and likewise the average ASL CBF and the DSC T_max_ perfusion lesion volumes were compared to the final infarct sizes respectively. A paired *t*-test was used to compare the mean lesion volumes between ADC and FLAIR imaging. The repeated measures analysis of variance (ANOVA) was used to examine the differences of mean lesion volumes among ASL CBF, T_max_ >4 s, T_max_ >5 s, T_max_ >6 s and FLAIR maps. The least significant difference (LSD) post hoc test was used to determine which pair comparisons were significantly different. All statistical tests were 2-tailed and a *p* value of <0.05 was considered significant.

## Results

**Figure 1:**
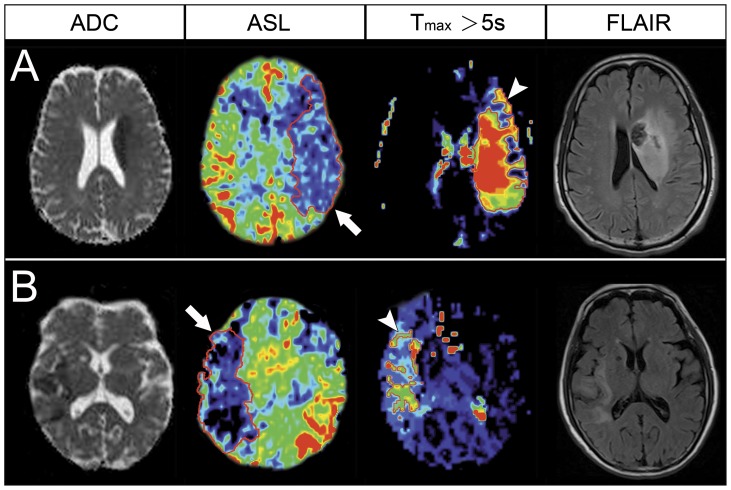
ADC, ASL perfusion (CBF), DSC perfusion (T_max_ >5 s), and follow-up FLAIR imaging of the representative patients. (A) A 60-year-old man underwent MRI at 8.8 hours after stroke onset. The ADC imaging showed an acute infarct in the left MCA territory. Larger perfusion defect areas were seen in the ASL map (arrow) and T_max_ map (arrowhead), indicating a mismatch. The follow-up FLAIR imaging showed a progressed infarct with hemorrhage. (B) A 77-year-old man underwent MRI at 8.2 hours after stroke onset. The ADC imaging showed an infarct in the right MCA territory. Although a large perfusion defect was observed in the ASL map (arrow), the Tmax map showed only a small defect (arrowhead), consistent with the ADC and follow-up FLAIR imaging.

**Figure 2:**
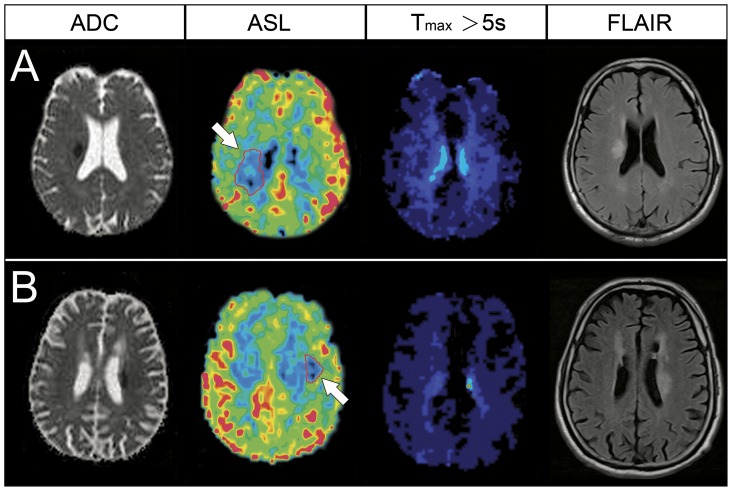
ADC, ASL perfusion (CBF), DSC perfusion (T_max_ >5 s), and follow-up FLAIR imaging of the representative patients. (A) A 76-year-old man underwent MRI at 5.0 hours after stroke onset. An acute infarct was noted in the right centrum semiovale in the ADC and follow-up FLAIR images. A perfusion defect was observed in the ASL map (arrow) but not in the T_max_ map. (B) A 60-year-old man underwent MRI at 22.6 hours after stroke onset. The ADC image showed an acute infarct in the left centrum semiovale with a corresponding perfusion defect in the ASL map (arrow), but not in the T_max_ map.

The 39 patients in this study (22 women, 17 men; mean age of 72.6±11.4 years, range 36–89 years) underwent DSC and ASL MR perfusion scans within 30 hours after onset of symptoms. The demographic details of the patient and perfusion results are shown in Table 1 (Group 1). Figures 1 and 2 show representative patients with ADC, ASL perfusion (CBF), DSC perfusion (T_max_ >5 s), and follow-up FLAIR imaging.

**Table 1:** Demographic data of clinical information and perfusion analysis (Mean±SD).

	Group 1* N = 39	Group 2# N = 31
Gender (F/M)	22/17	16/15
Age	72.6±11.4	71.7±11.2
NIHSS	8.4±6.9	7.5±5.6
Time to MRI (hr)	13.9±7.2	14.7±7.7
ADC volume (ml)	11.7±23.1	8.8±15.4
ASL volume (ml)	50.1±56.4	49.6±57.5
DSC volume (T_max_>4s) (ml)	39.0±62.0	36.2±61.2
DSC volume (T_max_>5s) (ml)	29.2±49.8	26.9±49.6
DSC volume (T_max_>6s) (ml)	21.8±39.0	19.9±38.9
Final infarct(FLAIR) (ml)		18.1±26.0

* Group 1: patients underwent DSC/ASL perfusion.

#Group 2: patients underwent DSC/ASL perfusion and follow-up image.

The ICC of the ASL CBF lesion volumes measured by the 2 readers was 0.96 (*p* <0.001), indicating a high reliability. The ASL CBF lesion volume showed significant correlation with the DSC lesion volume for T_max_ >4 s (*r* = 0.81, *p*<0.001), T_max_ >5 s (*r* = 0.82, *p*<0.001) and T_max_ >6 s (*r* = 0.80, *p*<0.001). However, the mean ASL CBF lesion volume (50.1 ml) was significantly larger than DSC lesion volume for T_max_ >5 s (29.2 ml, *p* = 0.002) and T_max_ >6 s (21.8 ml, *p*<0.001) but it did not reach a significant difference for T_max_ >4 s (39.0 ml, *p* = 0.42) as analyzed by the repeated measures ANOVA and LSD post hoc test.

**Figure 3:**
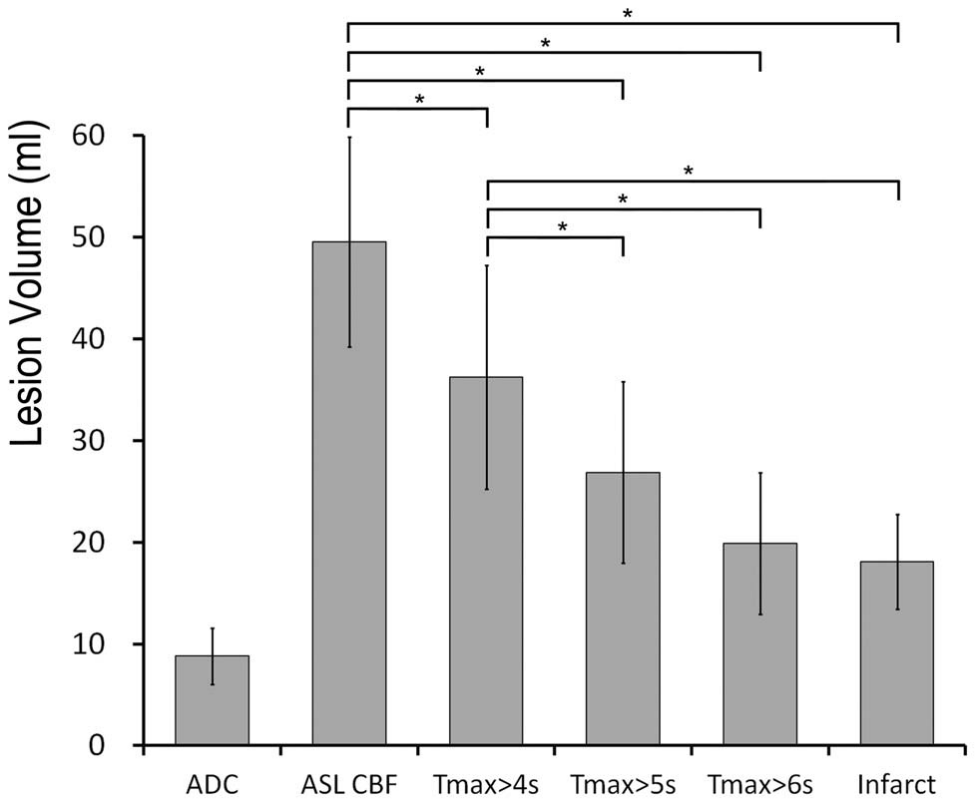
The mean lesion volumes of ADC, ASL CBF, DSC T_max_(>4–6 s), and final infarct in 31 patients with follow-up image. Bars indicate as mean±standard error. *: Statistically significant, p<0.05, by repeated measures ANOVA & LSD post hoc test.

Among 39 patients, 31 underwent follow-up MRI scans at 8.3±2.9 days after the first-time MRI (Table 1, group 2). The mean ADC lesion volume of 31 patients became larger in the follow-up FLAIR imaging (8.8±15.4 ml vs. 18.1±26.0 ml, *p* = 0.001). The mean lesion volume in ASL CBF was significantly larger than those in DSC T_max_ (>4–6 s, *p*<0.05) and FLAIR imaging (*p*<0.05), as analyzed by the repeated measures ANOVA and LSD post hoc test (Fig. 3). The mean lesion volume in DSC T_max_>5 s and 6 s was not different from final infarct in FLAIR imaging, indicating T_max_>5 s and 6 s might be close to the final infarct. The DSC lesion volumes measured with the T_max_ maps correlated well with the final infarct volume (*r* = 0.94, *r* = 0.95, and *r* = 0.94 for 4, 5, and 6 s delay; *p*<0.001). The ASLCBF lesion volume was also correlated to the final infarct volume (*r* = 0.86, *p*<0.001).

**Figure 4:**
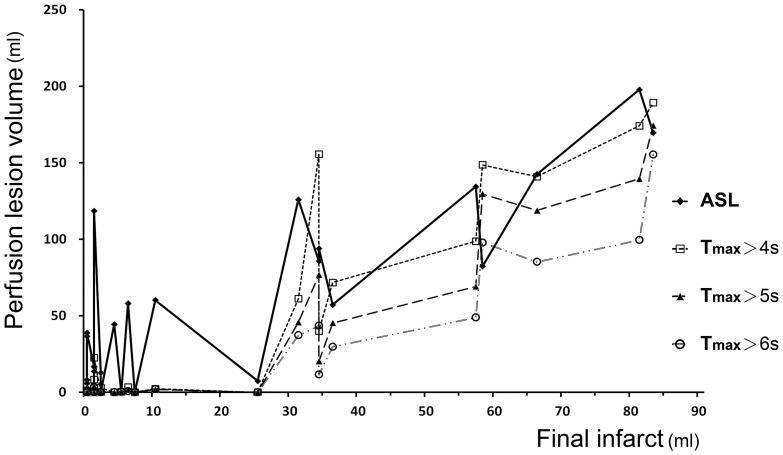
ASL and DSC lesion volumes with correlation to the final infarct volumes. Compared to DSC, ASL usually overestimated the perfusion deficits, especially in patients with small final infarcts.

Figure 4 demonstrates a graph of ASL and DSC lesion volumes along with correlation to the final infarct volumes. The perfusion lesion volume was larger in the ASL CBF maps than in the DSC T_max_ maps. Of the patients with final infarcts smaller than 30 ml, there were 9 with mismatches greater than 10 ml between ASL and DWI lesion volumes (ASL lesion 20.3±29.3 ml; ADC lesion 1.9±1.9 ml). However, these mismatches were not detected in the DSC perfusion maps (T_max_>5 s). The ASL volume was not correlated with the final infarct in these patients (*r* = 0.06, *p* = 0.78).

## Discussion

In conclusion, our study demonstrated that quantitative measurement of ASL perfusion in acute stroke correlates with DSC perfusion. However, ASL perfusion tended to overestimate the penumbra, especially in patients with small infarct size. Further refinement of the true penumbra threshold and improved ASL technique are necessary before applying ASL perfusion MRI in therapeutic trials.

There were many studies try to define the area of the penumbra between the infarct core and the benign oligemia. Although T_max_ of >2 s has been applied in previous clinical trials for penumbral selection [2], [3], [4], it was found to overestimate the penumbra. Further studies have suggested that T_max_ of >4–6 s might provide a more realistic estimate [21], [22], [23]. T_max_ of >6 s was used in the DEFUSE-2 study [24] and the forthcoming EXTEND trial [25]. Hence, we selected T_max_ of 4–6 s lag to estimate the perfusion defects. Our study showed that the ASL CBF lesion volume correlates well with the DSC T_max_ lesion volume for 4, 5, and 6 s delay. The lesion volumes in ASL and DSC perfusion imaging were all correlated to the final infarct volumes. The T_max_ perfusion map of >5 s had highest correlation with final infarct and the mean lesion volumes for T_max_>5 s and 6 s were not different from the final infarct, presumably T_max_ of >5 s or >6 s closing to true penumbra threshold. However, the mean lesion volume in the ASL maps was significantly larger than those of T_max_ >5 s and 6 s. These findings suggest that ASL perfusion imaging may overestimate the true penumbra. This is similar to the results of Zaharchuk et al. who found that ASL had a high negative predictive value for excluding mismatch but a low positive predictive value for DSC PWI and DWI mismatch (T_max_ >6 s) [12].

In our study, ASL tended to overestimate the perfusion defect volume in patients with a small final infarct. In the patients with final infarcts of <30 ml (Fig. 3), the DSC T_max_ perfusion maps did not detect any mismatches in the 9 patients who had mismatches of >10 ml between the ASL and ADC volumes. The ASL lesion volume was not correlated with the final infarct volume in these patients. The discrepancy may be due to the overestimation of mild perfusion defect by visual inspection, delayed arterial transit time or poor signal in white mater. Hence, a stricter ratio for relative CBF or a cut-off point for absolute CBF may be needed to define the true penumbra, rather than visual assessment only. In contrast, the ASL perfusion volumes were smaller than DSC volumes for 4 patients with large perfusion abnormalities (Fig. 4). In these four patients, the large perfusion defects were overlapped with the border zone sign, which was a bilateral ASL signal loss in the border zones resulting from increased arterial transit time [26]. The signal in the border zone area was same to the contralesional hemisphere. We did not choose these areas and this caused the underestimation of perfusion defect by ASL measurement. Because the border zone sign due to increased arterial transit time is common in old age and cerebrovascular diseases, improved ASL methodologies is necessary especially in this situation.

A potential limitation of this study was that no CBF threshold other than visual assessment was used to evaluate the penumbra in ASL perfusion. Areas of benign oligemia may be included in the penumbra, possibly resulting in poor reproducibility and limiting its application in clinical trials. Hence, an automated measurement with comparison to contralateral areas should be used to assess the CBF threshold defining the true penumbra. Besides, due to the limited sequence availability in our scanner, pulsed ASL pulse sequence (Q2TIPS) was used in this study. This sequence has been applied effectively in previous study [20], [27], [28]. However, this is a relatively old ASL technique with low signal-to-noise ratio (SNR). Additionally, a major problem with using ASL perfusion to measure the absolute CBF is its insensitivity towards white matter (WM) [29]. Some recently developed ASL techniques may provide better imaging quality and contrast, such as background suppressed, pseudo-continuous ASL with 3-T MRI, but deep WM signals remain unsatisfactory [30]. The ASL technique also tends to underestimate cerebral perfusion due to delayed blood flow since ASL perfusion acquires the signal at a fixed time point after labeling. Therefore, there may be discrepancies in areas with markedly long arterial transit times, such as patients with border zone sign. This problem may be minimized by the recently developed velocity-selective ASL technique [31] but the SNR was not good enough.

Another limitation of this study was that we compared 2 different modalities: CBF in ASL and T_max_ in DSC. Although there were consistent associations between ASL CBF and DSC T_max_ [6], the results may have been different if other parameters were chosen. In addition, our study was limited by the small number of cases, in regard to heterogeneous data in the onset time, infarct size, and stroke subtypes. In the future, a large, prospective cohort study of hyperacute stroke is needed to validate ASL perfusion.

Although ASL can provide absolute quantitation of CBF, there are still some issues that need to be concerned, such as CBF underestimation, sensitivity in transit time and poor SNR. These limitations may also lead to the inaccuracy of ASL in comparison to DSC. Thus, improved ASL technique is necessary before applying it for penumbral selection in acute stroke.
